# Proteomic Approach for Comparative Analysis of the Spike Protein of SARS-CoV-2 Omicron (B.1.1.529) Variant and Other Pango Lineages

**DOI:** 10.3390/proteomes10040034

**Published:** 2022-10-14

**Authors:** Mukul Jain, Nil Patil, Darshil Gor, Mohit Kumar Sharma, Neha Goel, Prashant Kaushik

**Affiliations:** 1 Parul Institute of Applied Sciences, Parul University, Vadodara 391760, Gujarat, India; nil.patil22636@paruluniversity.ac.in (N.P.); darshilgor150@gmail.com (D.G.); 2 Lab 209—Cell & Developmental Biology, Centre of Research for Development, Parul University, Vadodara 391760, Gujarat, India; 3 School of Molecular Medicine, Medical University of Warsaw, ul. Żwirki i Wigury 61, 02-091 Warsaw, Poland; ksmohit@gmail.com; 4 Malopolska Center of Biotechnology, 30-387 Krakow, Poland; 5 Institute of Biomedicine, University of Turku, 20500 Turku, Finland; nehagoel24march@gmail.com; 6 Independent Researcher, 46022 Valencia, Spain

**Keywords:** spike, Omicron, ACE2, SARS-CoV-2

## Abstract

The novel SARS-CoV-2 variant, Omicron (B.1.1.529), is being testified, and the WHO has characterized Omicron as a variant of concern due to its higher transmissibility and very contagious behavior, immunization breakthrough cases. Here, the comparative proteomic study has been conducted on spike-protein, hACE2 of five lineages (α, β, δ, γ and Omicron. The docking was performed on spike protein- hACE-2 protein using HADDOCK, and PRODIGY was used to analyze the binding energy affinity using a reduced Haddock score. Followed by superimposition in different variant-based protein structures and calculated the esteem root mean square deviation (RMSD). This study reveals that Omicron was seen generating a monophyletic clade. Further, as α variant is the principal advanced strain after Wuhan SARS-CoV-2, and that is the reason it was showing the least likeness rate with the Omicron and connoting Omicron has developed of late with the extreme number of mutations. α variant has shown the highest binding affinity with hACE2, followed by β strain, and followed with γ. Omicron showed a penultimate binding relationship, while the δ variant was seen as having the least binding affinity. This proteomic basis in silico analysis of variable spike proteins of variants will impart light on the development of vaccines and the identification of mutations occurring in the upcoming variants.

## 1. Introduction

The SARS-CoV-2 virus is the causative agent of COVID-19, which consists of single-stranded RNA and is enveloped with proteins [1]; it was defined as a global pandemic disease by WHO in 2019 and is a virus that causes respiratory tract and gastrointestinal infections in humans (host) [2]. Due to the high mutation rate, the variants in the virus were characterized as Variants of Concern and Variants of Interest. Based on genetic changes in spike protein, the Variant of Concern includes five major variants α SARS-CoV-2, β SARS-CoV-2, γ SARS-CoV-2, δ SARS-CoV-2 and Omicron strain (B.1.1.529) [3]. Omicron SARS-CoV-2 strain was highly responsible for pandemic conditions around the globe. It was more widely mutated in spike genes than any other previous strains [4]; these mutations in the spike gene directly influence the structure and function of spike protein and cause an aggressive stage of the disease. Because spike proteins are responsible for host-pathogen interaction [5]. The spike protein in SARS-CoV-2 has two subunits (S1 and S2). S1 contains a receptor binding domain (RBD) on the N terminus that serves to bind with the receptor. At the same time, S2 has a fusion peptide with two heptad-repeat domains (HR1, HR2) on C-terminus whose function is to enter and destabilize the host cell membrane [6,7]. The host cell has receptors such as hACE2(Antagonistic converting enzyme), C- type lectins, TIM1 (T cell immunoglobulin mucin domain-1), TAM (Targeting Tyro3, Axl, and MerTK), AXL (Anexeletkto), CD147 (Cluster of differentiation 147) and TMPRSS-2 (Transmembrane protease, serine 2.) which aggravate the entry of SARS-CoV-2 [8]. hACE2 is an enzyme that occurs on the cell membrane type II alveolar cells (lungs), enterocytes (small intestine), and endothelial cells (arteries and veins) and serves as host cell membrane receptor and primary target for SARS-CoV-2 [9,10]. The interaction between RBD of the S1 protein and hACE2 is the early stage of SARS-CoV-2 infection in the host. In this interaction, 20 residues of hACE2 and 17 residues of RBD result in the formation of a hydrophilic side-chain interaction [11]. Thirty mutations, 15 of which occur in the receptor-binding domain, as well as three tiny deletions and one minor insertion, dictate the spike protein’s variation [12]. In this present investigation, using different *In-silico* tools, we identified the variability in sequence, structure, mutational study and pathogenicity of spike protein (Omicron) with the existing strains of SARS-CoV-2. Comparison of transmissibility with the host cell, which is resulted by the interaction between humanACE2 and spike protein (α coronavirus, β coronavirus γ coronavirus, and δ coronavirus and omicron coronavirus were identified by molecular docking. Our current study would open new avenues for identifying unpredicted mutations responsible for host-pathogen interaction [13].

## 2. Materials and Methods

### 2.1. Data Collection

The protein sequences human receptor hACE2, α, β, δ, γ and Omicron were retrieved from GenBank NCBI (Accession ID: AB046569; 2581 bp, Accession ID: OM189517; 3061 bp, Accession ID: PA544048; 7033 bp, Accession ID: OM858819; 3813 bp, Accession ID: OM189519; 3361 bp, Accession ID: OM858820;3781 bp) respectively. The X-ray crystallography based protein template structure were retrieved for human receptor hACE2 (PDB ID: 7WBL [14]) and SARS-CoV-2 spike protein of different variants (α PDB ID: 7CYD [15], β PDB ID: 7VX1 [16], δ PDB ID: 7W92 [17], γ PDB ID:6XS6 [18] and omicron PDB ID: 7T9J [19]) from protein data bank (https://www.rcsb.org, accessed on 20 August 2022) with parameters such resolution 3.4 Å,3.55 Å,3.50 Å, 3.10 Å, 3.7 Å and 2.79 Å respectively.

### 2.2. Determination of Physicochemical Properties

The physical and chemical characteristics, such as molecular weight, several amino acids, aliphatic index, theoretical pI, instability index, and grand average of hydropathy (GRAVY) [20] of the SARS-CoV-2 and other variants spike proteins, were computed through Expasy ProtParam tool (https://web.expasy.org/protparam, accessed on 21 August 2022).

### 2.3. Prediction of Immunoproperties

The ABCpred server (https://webs.iiitd.edu.in/raghava/abcpred/ABC_submission.html, accessed on 22 August 2022) was used to predict B-cell epitopes for SARS-CoV-2 variants [21]. ABCpred server utilizes the artificial neural network and calculates the sensitivity and specificity of amino acids having the correct probability of being in epitope regions; this server assigns a score of 0–1, where 1 represents the higher authenticity of the predicted epitope region and vice-versa. BepiPred 2.0 server (https://services.healthtech.dtu.dk/service.php?BepiPred-2.0, accessed on 22 August 2022) was used to anticipate the epitope of exposed B-cell [22]. The immunogenicity of T-cell epitopes was predicted through the IEBD Analysis Resource server (http://tools.iedb.org/main, accessed on 22 August 2022) [23]. NetCTL 4.0 server (https://services.healthtech.dtu.dk/service.php?NetCTLpan-1.1, accessed on 22 August 2022) is helpful to predict cytotoxic T-lymphocytes [17].

### 2.4. Phylogenetic Tree Construction and Primary Amino Acid Sequence Alignment

The α, β, δ, γ, and omicron SARS-CoV-2 spike protein sequences were retrieved in FASTA format from Protein Data Bank. Studies of the mutation in spike protein and increase in viral transmissibility were inferred by the evolutionary link of spike protein sequences through the phylogenetic tree [24]. Multiple sequence alignment has been done by using the MUSCLE approach with 1000 bootstrap and distance-based neighbor-joining (NJ) based phylogenetic tree construction for protein sequences generated in Molecular Evolutionary Genetics Analysis (MEGA-X) [25].

### 2.5. Comparative Analysis of the Secondary and Tertiary Structure of Omicron

The GOR (Garnier–Osguthorpe–Robson) tool employs information theory and Bayesian statistics for secondary protein structure analysis. The GOR IV was used to predict secondary structure α, β, γ, δ, and omicron variants [26,27]. Protein tertiary structure prediction has done using PDB templates for Omicron, α, β,γ, and δ SuperPose10.1 webserver (http://superpose.wishartlab.com, accessed on 25 August 2022) based on the eigenvalue matrix was used to analyze the pairwise structure alignment. SuperPose used a modified quaternion eigenvalue technique [28]. SuperPose is used to measure the maximum deviation in tertiary structures, RMSD data, as well as difference distance charts and values of the molecules superimposed, which are in numerical form. The technique of orienting an item until it can be immediately placed on top of another object is known as superposition or superimposition [29].

### 2.6. Protein-Protein Interactions

Protein-protein docking was performed between spike proteins of α, β, δ, γ, Omicron, and hACE2 with the help of the HADDOCK v2.4 server [30,31]. For docking purposes, blind docking was performed between variants and hACE2. For docking we have used folloing input parameters (Supplementary Table S2). In total, five docking runs were executed, and for every run, 10 clusters of four poses each were generated through the HADDOCK server. A further cluster with the least HADDOCK score was selected for their binding energy study via PRODIGY for all five docking runs. PRODIGY (PROtein BinDIng enerGY prediction) (https://wenmr.science.uu.nl/prodigy, accessed on 26 August 2022) is a set of online services aimed at predicting binding affinity in biological complexes and identifying biological interfaces based on crystallographic data [32]. Finally, the interacting residues of both chains, salt bridges, H-bonding between residues of two chains, and nonbonded interactions were calculated through PDBsum (http://www.ebi.ac.uk/thornton-srv/databases/cgi-bin/pdbsum, accessed on 26 August 2022). PDBsum is a visual database that shows the components within each three-dimensional structure deposited in the Protein Data Bank at a glance (PDB) [33]. 

## 3. Results

### 3.1. Physical Parameters of Proteins

In comparison to α, β, δ, γ and Omicron have the highest number of amino acids, 1116, 1258, 1261, 1256 and 1285, respectively. pI (isoelectric point) is the measure of pH at which the net charge of the surface is zero. As the pI of α (5.66) is far less than 7 indicates more acidic compared to the pI of Omicron, which is nearby 7, which is 6.63. Research shows that an instability index of less than 40 predicts that protein structure is stable; all variant II indicates that the spike protein shares stability. Aliphatic Index indicates the aliphatic amino acid present on the side chain of the concerned protein. A high AI indicates more thermal stability data indicating α is maximally thermostable, and Omicron is least thermostable compared to other variants. GRAVY indicates hydropathicity; the lower the score, the more would have an affinity toward the water; γ and Omicron show a stronger affinity towards water compared to others.

### 3.2. Prediction of Immune Properties

Exposed B cell epitope, which plays a vital role in antigen portion binding to the immunoglobulin interaction, varied from 33 to 40 (Table 1). Among all the variants, α and Omicron variants show equal scores for protective antigen (0.4646) and antigenicity (0.717053) (Table 1). Prediction for C–cell epitope ranged from 35 to 38. The Immunogenicity prediction scores for the spike protein variant are varied. The Omicron variant shows the highest number (27) of strong binders in T cells that extrapolate into an immunogenicity score of 0.49637, which means the omicron variant has more virulence transmissibility than other variants.

### 3.3. Comparative Sequences and Phylogenetic Analysis of Omicron Spike Protein

**Figure 1:**
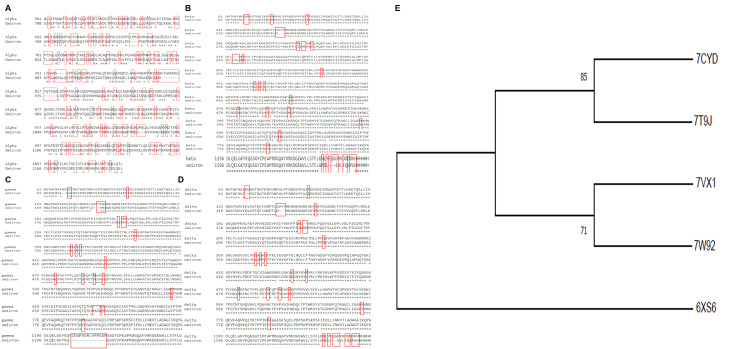
(**A**–**D**) Spike protein sequence analysis of α, β, δ, and γ variants concerning the omicron variant. Deletions, insertions, and mutations are marked with red blocks. (**E**) The phylogenetic tree was built by Molecular Evolutionary Genetics Analysis [MEGA-X]. The visualization of Omicron with α, β, δ, and γ was done through MAFFT. Numeric value denoting bootstrap value.

The branch length specifies genetic change, i.e., the extended branch and the additional genetic change (divergence) have happened. Omicron forms a sister group with β, showing the maximum divergence from α. Omicron is most diverged from other variants and has evolved lately with the greatest number of mutations. The percentage similarity between α and Omicron was (33.2%), β having a percentage similarity with Omicron of (94.9%), and δ when and γ were compared with Omicron, did not show much more significant differences in similarity. The percentage similarity between δ and Omicron is (95.2%), and between γ and Omicron is (95.1%) (Figure 1A,B). The amino acid substitution in Omicron compared to α, β, δ and γ variants were described in Supplementary Table S1.

### 3.4. Secondary and Tertiary Structure Analysis

Omicron spike protein has a 3.64%, 1.46%, 1.97% and 1.5% lower fraction of α-helix structure compared with spike proteins of α, β, δ, and γ. Spike protein of α has a higher extended strand of 5.88%, 6.32%, 5.05%, and6.3% compared with spike protein of β, δ, γ and Omicron, respectively. The Spike protein of Omicron has the highest deviation with α spike protein, around 9.93%, and the lowest deviation with β spike protein was about 1.97% (Table 2).

**Figure 2:**
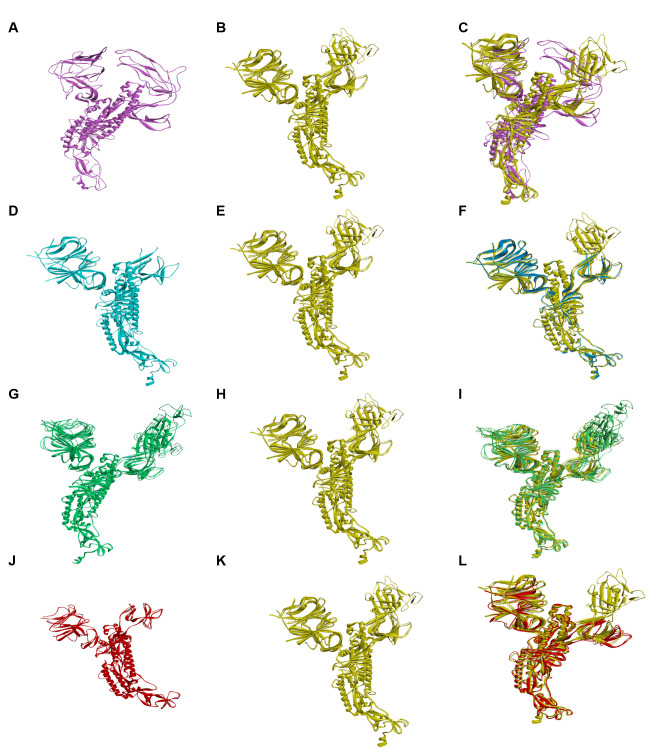
Secondary structure alignment of the spike protein of Omicron with spike protein of α, β, δ and γ variants. (**A**) Spike protein of α variant’s secondary structure, (**B**) spike protein of Omicron, (**C**) aligned secondary structure of α and omicron spike protein amino acids on 123 sites with 25 deletion sites. (**D**) Spike protein of β variant’s secondary structure, (**E**) Spike protein of omicron variant’s secondary structure, (**F**) aligned secondary structure of β and omicron spike protein amino acids on 25 sites with 12 deletion sites, (**G**) Spike protein of δ variant’s secondary structure, (**H**) Spike protein of omicron variant’s secondary structure, (**I**) aligned secondary structure of δ and omicron spike protein amino acids on 24 sites with 9 deletion sites, (**J**) Spike protein of γ variant’s secondary structure, (**K**) Spike protein of omicron variant’s secondary structure, (**L**) aligned secondary structure of γ and omicron spike protein amino acids on 15 sites with 25 deletion sites.

“SuperPose 10.1” is used to measure the maximum deviation in the tertiary structure of spike protein of Omicron with novel SARS-CoV-2 variants (Figure 2); these interactions provide the RMSD value of α-carbon, backbone and heavy chains in both local and global forms. An RMSD value in Angstrom, which represents measured RMSD between the superposed molecules, is one of the seven forms of output produced by SuperPose; this RMSD value is shown in two forms chain-wise and a whole structure in both forms, local and global (Table 3 and Table 4).

RMSD is mainly used for quantitative measurement (in angstrom) of the similarity between two superimposed atomic coordinates. As per the result, α-omicron (PDB_ID: 7CYD–7T9J) has the highest RMSD score between α-carbon around 2.785Å, backbone having 2.783Å and a heavy molecule having an RMSD score of 2.903Å; these high RMSD values denoted that the spike protein of Omicron has distinguished from the spike protein of α variant.

### 3.5. Proteome-Based Mutational Analysis of Spike Protein Domains

**Figure 3:**
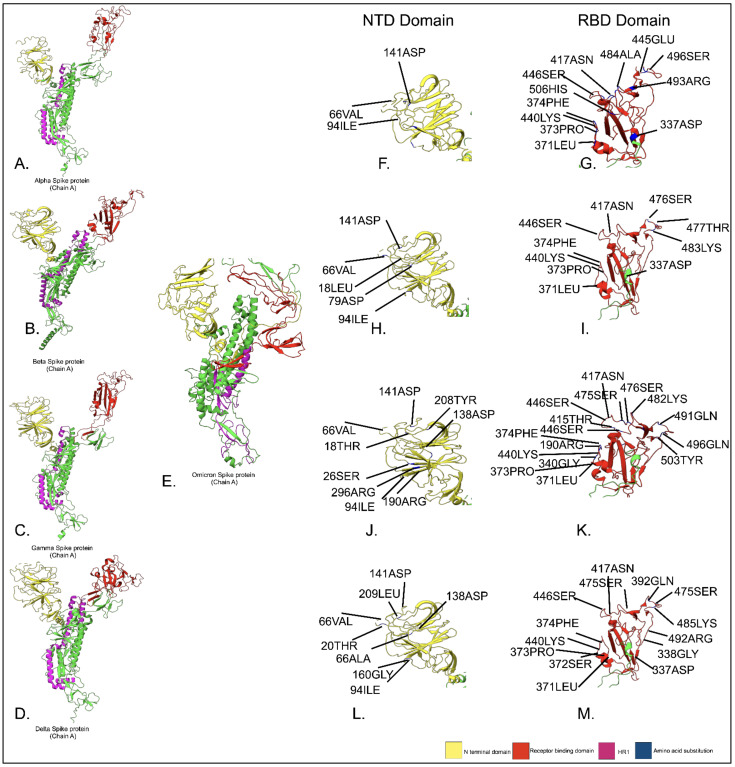
Comparative mutation analysis of NTD and RBD domain of omicron spike protein with respect to other pangolineages. (**A**) PDB structure of alpha variant spike protein (ChainA); (**F**) NTD domain of alpha variant spike protein depicting amino acid residue substitution in comparison to alpha variant NTD; (**G**) RBD domain of alpha variant spike protein depicting amino acid residue substitution in contrast to alpha variant RBD, (**B**) PDB structure of beta variant spike protein (ChainA); (**H**) NTD domain of beta variant spike protein depicting amino acid residue substitution in comparison to alpha variant NTD, (**I**) RBD domain of beta variant spike protein depicting amino acid residue substitution in comparison to alpha variant RBD, (**C**) PDB structure of gamma variant spike protein (ChainA); (**J**) NTD domain of gamma variant spike protein depicting amino acid residue substitution in comparison to alpha variant NTD, (**K**) RBD domain of gamma variant spike protein depicting amino acid residue substitution in comparison to alpha variant RBD (**D**) PDB structure of delta variant spike protein (ChainA); (**L**) NTD domain of delta variant spike protein depicting amino acid residue substitution in comparison to alpha variant NTD, (**M**) RBD domain of delta variant spike protein representing amino acid residue substitution in comparison to alpha variant RBD; (**E**) PDB structure of Omicron variant spike protein (ChainA).

The earlier data suggest that there was structural variation in the spike protein of the SARS-CoV-2 virus, and the spike protein of the α variant was highly deviated compared to the Omicron variant. Mutational analysis altered the amino acid in the spike protein’s domains (RBD and NTD). Alterations in the amino acid sequence of the RBD can significantly affect S binding affinity for hACE2 and, ultimately, SARS-CoV-2 infectivity. Although mutations occur throughout this region, direct interactions with potential ligands are still feasible because most of the mutations in this area are found on the surface of S [34] Figure 3. Deep mutational studies are being conducted to determine whether single-site mutations affect the hACE2 affinity in this region; these results might be contrasted with emerging concerns as of March 2022; the significant change in the NTD domain of Omicron in comparison to other variants (T94I, G141D, and A66V) is found in the beta variant (A79D) and in the gamma variant (Y138D). The major changes in the RBD domain of Omicron in comparison to other variants are (G337D, S371L, S373P, S374F, N440K, G446S, and S417N); these results suggest that the key mechanism driving the positive selection of mutations within the RBD is not the host receptor’s binding affinity for S. Furthermore, the majority of mutations in this area modify the RBD’s charge or hydrophobicity, greatly increasing the likelihood that the antibody may escape through altered epitope affinities or regional conformational changes that reduce epitope accessibility. Numerous factors, including widespread common mutations throughout the NTD subdomain, contribute to the positive selection of variants carrying mutations in the NTD of SARS-CoV-2 S. Although the NTD is the target of 35% of SARS-CoV-2 antibodies, only around one-third of these antibodies have a neutralizing impact [35].

### 3.6. Protein-Protein Interaction Analysis: (Spike-SARS-CoV-2)-hACE2

The binding of SARS-CoV-2 to the host receptor is a key factor in infectivity, transmission, and pathogenesis, hence alteration in the structure of the spike protein (NTD and RBD) domain during the evolution of the virus would have a significant impact on these processes. Using HADDOCK 2.4, protein-protein docking was executed between spike proteins of α, β, δ, γ, and Omicron, with human hACE2 (hACE2). The binding affinity was calculated through PRODIGY. The results in HADDOCK displayed the 10 best clusters, and the one with the lowest HADDOCK score was taken into account to calculate the binding affinity. As per the result, the Omicron variant shows the highest HADDOCK score and binding affinity (Table 5) compared to other variants. Further, the interaction analysis was done through PDBsum taking the cluster mentioned above for different dockings. 

It was observed in this study that in comparison to other variants of SARS-CoV-2. In Omicron, spike protein found 32 hydrogen interactions involving N417, Y449, Y453, L455 and N487 residues with hACE2. Additionally, the number of salt bridges increased from one to three when the RBDs of Omicron spikes protein bind with hACE2. Majorly the N501Y alterations, which were previously reported for the α variation, also boosted the binding affinity for the Omicron variant because the number of hydrogen bonds and Pi-Cation link were increased (Table 6 and Table 7). In addition, it was observed that mutations enhanced the binding affinity between the receptor-binding domain of spike protein and hACE2, which further elucidated the mutational changes in the RBD domain and increased the pathogenesis and transmission of the Omicron variant.

## 4. Discussion

There are various proteomics techniques available for the identification and which enable the study of the interaction between host proteins and virus spike proteins, to understand evolutionary lineages. Proteomics can be used to understand intricate SARS-CoV-2 interaction with the host cell. In this study, different types of computational approaches are used to compare different types of SARS-CoV-2 variants (α, β, γ, δ and Omicron) based on sequence, physiochemical properties, structure, and how they alter the interaction with host receptor protein hACE2. Different variants of SARS-CoV-2 show remarkable scores in terms of immunogenicity and antigenicity. Especially omicron variant showed high antigenicity and low exposed B-cell epitopes, which denote the strongest bonding with an epitope and indicate the highest transmissibility. As per earlier research, phylogenetic relationships are established between Omicron with other variants based on the distance matrix [36]. Using the UPGMA algorithm, the mapping of variable strains at different branches was generated per the rules of phylogenetic preparation [37]. This study established an inference that Omicron shares a monophyletic clade [38]. The sequence variation or the mutation rate establishes an omicron variant dissimilar to the α variant as analyzed by polyphyletic classification based on the Neighbor-Joining methodology (MEGA-X) [39]; this establishes a probability about the rate of single nucleotide polymorphism, which directly causes a change in sequence, structure, and function of omicron variants. After the determination of position in the phylogenetic tree, analysis of functional variability among proteomes of different variants by computational methods shows a change in the surface charge in omicron spike protein compared to other variants due to mutation, which directly results in the increment of hydrophobic residues; this increase enhances the stability of the omicron protein core [40,41], while the change in the amino acids of the omicron RBD region of spike protein in comparison to other lineages affects the immune response and also the vaccine (Ab) interaction [42,43]. The transition from Proline-603 to lysine-730, aspartic acid 655 to valine 782, aspartic acid 669 to glycine 796, and many more in Omicron in comparison to α increase the positive charge and hydrophobicity, which improves its binding with hACE2(due to negative charge of protein) and stability [44]. Secondary structure analysis displays an increase in an α helix as compared to a δ variant; a greater α helix provides conformation stability which enhances the transmissibility in the host [45]. Variation in the secondary structure directly correlated with the tertiary structure, which consists of variable domains regulating its binding with hACE2. Spike protein consists of the following domains distributed according to different positions of amino acids such as 14–305 residues (N terminal domain), 306–330 residues (C terminal domain), and 331–527 (Receptor binding domain).686–815 (S1/S2 cleavage),816- 911(fusion peptide), 912–984 (heptad repeat),1035–1147(Connector domain) [46]. Compared to the omicron structure, in α-coronavirus spike protein, there is a reduction in α helix in NTD and RBD domain, while in β-coronavirus, in RBD less α helix are present, γ structure is similar, but δ RBD consist earlier omicron strain. Earlier research signifies the importance of protein-protein interaction as spike protein molecular interaction with hACE2 for the access of the virus into the host cell [47]. The substitution in amino acids present in the spike protein RBD domain in different strains from α, β, γ, δ, and Omicron due to mutation increases the transmissibility and infectivity of the virus. The change in amino acids in the RBD domain, such as Leu455, Phe486, Glu493, Ser494, and Asn501 alter the binding of SARS-CoV-2 with the host cell [48]. The interaction study can study this dynamic nature through docking and binding energy. The greater the affinity of hACE2 and spike protein is dependent on the kD value; the smaller the value, the more affinity. Data suggest that Omicron has more affinity than β and γ, directly interrelating its infectivity. As we know, humoral immunity may not be as effective as T cells in preventing the emergence of new coronavirus infections. Other research has also shown that CD8+ T cells may often target a range of SARS-CoV-2 antigens and identify epitopes from different viral antigens through a series of combinations of T-cell receptors (TCRs), which are critical for viral clearance, long-term immunity, and memory for protection [49]. The competence of CD8+ T cells to prevent secondary infection Because of its high specificity and ability to elicit a potent immune response, the SARS-CoV-2 spike protein has been put the focus of vaccine development [50]. Particularly, the RBD region is frequently regarded as a crucial protein target for vaccine design and the creation of therapeutic neutralizing antibodies. In this study, T cell MHC class-1 epitopes predicted which can evaluate the affinity between peptide and MHC molecule, which can infer in future vaccine development. Then predicted qualitative affinity physical and chemical properties and further studied immunogenic peptides for vaccine designing. In this study, we focused on sequence changes that occurred in the spike protein of Omicron; those changes will affect the binding of protein-based vaccine, an explanation that was useful in vaccine development and designing. The first protein-based vaccine was NuvaxovidTM (NVX-CoV2373) (Novavax Inc., Gaithersburg, MD, USA), which comprises the full-length S protein and possesses common epitopes that may be able to protect against all SARS-CoV-2 virus strains [51]. Anhui Zhifei Longcom/Chinese Academy of Medicine (ZF2001) (Anhui Zhifei Longcom, China), COVAXX/United Biomedical Inc. (UB-612), and Clover Biopharmaceuticals/GSK/Dynavax are three other examples of protein-based vaccinations (SCB-2019) [52]. Protein identification, quantification, protein-protein interactions, protein changes, and localization can all be studied using proteomics’ tools, and it is a part of proteomic complexity. Understanding the interaction between one protein from the SARS-CoV-2 virus (SPIKE) and one from *Homo sapiens* (ACE 2) opens new pavement to answer an unanswered question of protein complexity related to the interaction between viruses and humans. In summary, our analysis shows that in different corona cases, Omicron induced greater affinity with human Ace2 compared to non-Omicron SARS-CoV-2.

## 5. Conclusions

The comparative analysis of omicron spike protein based on the hydropathy index with other variants would open up new pavements in research. Simultaneously, the greater part of the mutations in the spike protein of the omicro-hACE2 interface appears to diminish hACE2 cooperation liking and may affect the binding interactions of upcoming variants; this is conceivably emerging from choice strain to work with invulnerable departure, as an impressive number of antibodies focus on a similar connection point. This study will also impart light on the developmental program of vaccines and the identification of mutations occurring in the spike protein of the upcoming variants.

## Figures and Tables

**Table 1:** Comparison of the immunological properties of the spike protein of SARS-CoV-2 variants.

Variants of SARS-CoV-2	Exposed B Cell Epitopes	Predicted Probability of Antigenicity Score	Number of Epitopes Identified in CTLa	Protective Antigen Prediction Score	Number of Strong Binders in T-Cell	Immunogenicity Predication Score
α (7CYD)	40	0.717053	37	0.4646	20	0.3019
β (7VX1)	40	0.643558	38	0.4542	09	1.23216
δ (7W92)	38	0.744007	35	0.4709	23	0.0304
γ (6XS6)	41	0.596261	34	0.4583	22	1.07515
Omicron (7T9J)	33	0.717053	35	0.4646	27	0.49637

**Table 2:** Deviation in the secondary and the tertiary structure of omicron spike protein compared to other SARS-CoV-2 variants through GORIV.

Parameters	α	β	δ	γ	Omicron
α helix (Hh)	262 is 23.4%	268 is 21.30%	275 is 21.81%	268 is 21.34%	255 is 23.46%
310 helix (Gg)	0 is 0.00%	0 is 0.00%	0 is 0.00%	0 is 0.00%	0 is 0.00%
Pi helix (Ii)	0 is 0.00%	0 is 0.00%	0 is 0.00%	0 is 0.00%	0 is 0.00%
β bridge (Bb)	0 is 0.00%	0 is 0.00%	0 is 0.00%	0 is 0.00%	0 is 0.00%
Extended strand (Ee)	290 is 25.99%	253 is 20.11%	248 is 19.67%	263 is 20.94%	253 is 19.69%
β turn (Tt)	0 is 0.00%	0 is 0.00%	0 is 0.00%	0 is 0.00%	0 is 0.00%
Bend region (Ss)	0 is 0.00%	0 is 0.00%	0 is 0.00%	0 is 0.00%	0 is 0.00%
Random coil (Cc)	564 is 50.54%	737 is 58.59%	738 is 58.52%	725 is 57.72%	777 is 60.47%
Ambiguous states (?)	0 is 0.00%	0 is 0.00%	0 is 0.00%	0 is 0.00%	0 is 0.00%
Other states	0 is 0.00%	0 is 0.00%	0 is 0.00%	0 is 0.00%	0 is 0.00%

**Table 3:** The maximum deviation in the tertiary structure of omicron spike protein with novel SARS-CoV-2 variants.

Interaction	RMSD	α Carbon	Backbone	Heavy	All
α–Omicron (7CYD–7T9J)	Local	2.785	2.783	2.903	2.903
	Global	2.785	2.783	2.903	2.903
β–Omicron (7VX1–7T9J)	Local	0.738	0.747	0.91	0.91
	Global	0.738	0.747	0.91	0.91
γ–Omicron (6XS6–7T9J)	Local	0.818	0.833	1.05	1.05
	Global	0.818	0.833	1.05	1.05
δ–Omicron (7W92–7T9J)	Local	1.437	1.438	1.673	1.673
	Global	1.437	1.438	1.673	1.673

**Table 4:** The maximum deviation in the tertiary structure of omicron spike protein’s different chains with novel SARS-CoV-2 variants.

Interaction	Chain	RMSD Value	α Carbon	Backbone	Heavy	All
α–Omicron (7CYD–7T9J)	A chain	Local	-	-	-	-
		Global	-	-	-	-
	B chain	Local	-	-	-	-
		Global	-	-	-	-
	C chain	Local	1.70	1.73	2.05	2.05
		Global	20.47	20.43	20.55	20.55
β–Omicron (7VX1–7T9J)	A chain	Local	43.60	43.59	43.48	43.48
		Global	43.60	43.59	43.48	43.48
	B chain	Local	44.04	44.03	43.89	43.89
		Global	44.04	44.03	43.89	43.89
	C chain	Local	64.35	64.35	62.23	62.23
		Global	64.35	64.35	62.23	62.23
γ–Omicron (6X6S–7T9J)	A chain	Local	2.39	2.40	2.64	2.64
		Global	2.39	2.40	2.64	2.64
	B chain	Local	2.22	2.24	2.46	2.46
		Global	2.22	2.24	2.46	2.46
	C chain	Local	2.41	2.43	2.63	2.63
		Global	2.41	2.43	2.63	2.63
δ–Omicron (7W92–7T9J)	A chain	Local	5.25	5.13	5.43	5.43
		Global	4.57	4.57	4.75	4.75
	B chain	Local	0.98	1.00	1.43	1.43
		Global	2.83	2.84	3.12	3.12
	C chain	Local	1.28	1.32	1.63	1.63
		Global	15.10	15.09	15.20	15.20

**Table 5:** The interaction analysis of spike protein of SARS-CoV-2 variants with hACE2 through PRODIGY.

Interaction of SARS-CoV-2 Variant’s Spike Protein with hACE2	Binding Affinity in kcal/mol
spike protein of α-hACE2	−10.8
spike protein of β-hACE2	−10.5
spike protein of δ-hACE2	−8.3
spike protein of γ-hACE2	−9.5
spike protein of omicron-hACE2	−11.8

**Table 6:** List of interactive residues of spike RBD residue of different variants of SARS-CoV-2 and hACE2 residues.

Interacting Proteins	Variants				
	Alpha	Beta	Gamma	Delta	Omicron
Spike-RBD residues	R403, Y453, A475, G485, F486, N487, C488, Y489, Q493, Q498, T500, N501, Y505	R408, T415,G416,N417,Y449, L452,Y453,L455,F456, A475, G476,T478,K484,F489,N487,Y489,Q493, G496, Q498,T500,Y501,G502,Y505	E329, K353, D405, T417, L455, F456, K484, F486, Q498, T500,Y501, Y505	R403, Y453, A475, G485, F486, N487, C488, Y489, Q493, Q498, T500, N501, Y505	N417, Y449, Y453, L455, F456, F486, N487, Y489, F490, R493, S494, S496, Y501
ACE2 residues	I21, Q24, K31, H34, D38, L39, Q42, M82, Y83, P84, E87	S19, Q24, T27,F28,D30,K31,H34,E35,D38,Y41,Q42,L45,L79,M82, Y83	Y41, D30, E35, E37, D38, L39, Q42, M82, Y83, P84, E87	I21, Q24, K31, H34, D38, L39, Q42, M82, Y83, P84, E87	T27, F28, D30, K31, H34, E35, D38, T78, L79, M82, K353

ACE2: Angiotensin-converting enzyme 2; RBD: Receptor binding domain.

**Table 7:** Protein–protein docking of α-hACE2, β-hACE2, δ-hACE2, γ-hACE2 and Omicron-hACE2 interaction analysis through PDBsum showing the number of H-bonding and Salt bridges.

SARS-CoV-2 Variant’s Spike Protein -hACE2 Interaction	Chain A (Spike-Variant) Residues	Chain B (hACE2) Residues	Salt Bridges	H-Bonding	Non-Bonded Contacts
α-hACE2	10	15	1	7	77
β-hACE2	18	11	2	16	67
δ-hACE2	18	12	1	8	73
γ-hACE2	16	16	2	8	113
Omicron-hACE2	12	17	3	32	74

## Data Availability

Data are made available via computational bioinformatics tools. There are various bioinformatics tools used by the authors to compare spike protein sequence, structure of different strains of SARS-CoV-2, and comparison with its affinity with hACE2 (Human). For protein data we used Protein Data Bank https://www.rcsb.org (accessed on 20 August 2022), For immunological prediction we used ABCpred server https://webs.iiitd.edu.in/raghava/abcpred/ABC_submission.html (accessed on 22 August 2022) BepiPred 2.0 server https://services.healthtech.dtu.dk/service.php?BepiPred-2.0 (accessed on 22 August 2022) IEBD Analysis Resource server http://tools.iedb.org/main (accessed on 22 August 2022) NetCTL 1.0 server https://services.healthtech.dtu.dk/service.php?NetCTLpan-1.1 (accessed on 22 August 2022). For phylogenetic tree construction https://www.megasoftware.net (accessed on 24 August 2022). MAAFT V7.0 https://mafft.cbrc.jp/alignment/server (accessed on 24 August 2022). GOR IV https://npsa-prabi.ibcp.fr/NPSA/npsa_gor4.html (accessed on 25 August 2022). SuperPose 10.1 http://wishart.biology.ualberta.ca/SuperPose (accessed on 25 August 2022). For protein protein docking and their analysis we used HADDOCK v2.4 server https://wenmr.science.uu.nl/haddock2.4 (accessed on 26 August 2022) PRODIGY https://wenmr.science.uu.nl/prodigy (accessed on 26 August 2022) PDBsum http://www.ebi.ac.uk/thornton-srv/databases/cgi-bin/pdbsum (accessed on 26 August 2022).

## Supplementary Materials

The following supporting information can be downloaded at: https://www.mdpi.com/2227-7382/10/4/34/s1, Table S1: The amino acid substitution in Omicron compared to α, β, δ and γ variants. Table S2. Parameter for HADDOCK 2.4.
