# Correction: Jain et al. Proteomic Approach for Comparative Analysis of the Spike Protein of SARS-CoV-2 Omicron (B.1.1.529) Variant and Other Pango Lineages. *Proteomes* 2022, *10*, 34

**DOI:** 10.3390/proteomes12030019

**Published:** 2024-07-09

**Authors:** Mukul Jain, Nil Patil, Darshil Gor, Mohit Kumar Sharma, Neha Goel, Prashant Kaushik

**Affiliations:** 1 Parul Institute of Applied Sciences, Parul University, Vadodara 391760, Gujarat, India; 2 Lab 209—Cell & Developmental Biology, Centre of Research for Development, Parul University, Vadodara 391760, Gujarat, India; 3 School of Molecular Medicine, Medical University of Warsaw, ul. Żwirki i Wigury 61, 02-091 Warsaw, Poland; 4 Malopolska Center of Biotechnology, 30-387 Krakow, Poland; 5 Institute of Biomedicine, University of Turku, 20500 Turku, Finland; 6 Independent Researcher, 46022 Valencia, Spain

## Affiliation Correction

In the publication [1], there was an error regarding the affiliation for “Prashant Kaushik”. The updated affiliation should be: “Independent Researcher, 46022 Valencia, Spain”.

The authors state that the scientific conclusions are unaffected. This correction was approved by the Academic Editor. The original publication has also been updated.
