# Supplementary material for: Proteomic Approach for Comparative Analysis of the Spike Protein of SARS-CoV-2 Omicron (B.1.1.529) Variant and Other Pango Lineages
Source: Proteomes. 2022 Oct 14;10(4):34. doi: 10.3390/proteomes10040034 (PMC9624331; doi:10.3390/proteomes10040034)
Supplement: Supplementary file 1 [file proteomes-10-00034-s001.zip › proteomes-1890546-supplementary.pdf]

Supplementary Material:

Table S1. The amino acid substitution in omicron compared to α, β, δ & γ variants.

	α Variant	β Variant	δ Variant	γ Variant
**Compared to Omicron variant changes occurred in Amino Acid residues**	S583-A710; T590-I717; V594-T721; Q598-P725; S601-M728; P603-K730; I604-T731; V605-S732; T610-M737; N614-G741; G615-D742; V617-T744; V620-S747; K624-L751; T627-G754; N639-G766; S640-I767; L643-E770; A646-K773; V648-T775; T653-A780; F654-Q781; D655-V782; L661-T788; N663-P790; S666-Y793; D669-G796; A687-S810; G688-K811; V701-T824; T702-L825; S703-A826; G704-D827; T707-F830; K713-G835; T716-L838; K717-G839; G718-D840; S720-A842; A722-R844; Y729-K851; M734-T856; A742-D864; R744-M866; M747-Q869; G750-S872; S751-A873; I756-T878; L758-T880; G759-S881; S763-F885; S766-G888; S770-P896; A772-Q898; Q774-A900; S775-Y901; Q788-Y914; A796-N922; T803-G929; V806-Q932; A808-S934; V826-T938; N851-G943; S843-A955; T848-V960; Q856-G968; S961-V973; A864-D976; Q876-V988; A887-Q999; A888-S1000; V891-T1003; T898-I1010; T901-A1013; R907-A1019; Q911-A1023; K919-L1031; S920-G1032; G926-D1038; T932-Y1044; A940-S1052; T950-V1062; V951-T1063; T955-A1067; Y957-E1069; A962-T1074; S964-P1076; V968-H1080; N972-A1084; G973-H1085; L976-R1088; Q978-G1090; Y989-H1098; R991-F1100; I996-N1105; I1005-T1114; A1006-D1115; F1008-T1117; Q1010-V1119; I1011-S1120; E1012-G1121; T1017-V1126; N1020-I1129; E1025-V1134; T1028-P1137; V1030-Q1139; I1034-D1143; S1044-F1153; Y1045-K1154; L1047-H1156; P1048-T1157; P1053-D1162; D1054-L1163; V1056-D1165; Q1059-G1168; Q1062-A1171; K1077-V1186; S1078-A1187; A1079-K1188; Y1083-E1192; Q1086-I1195	G142-D140;N211-I206;G217-D212; I243-246R; G336D; S368L; S370P; S372F; G443S; K481A; G493S; Y502H; H652Y; P678H; D793Y; E1235G; L1237S; K1244L; D1245F; D1247G; D1248P; K1249G,	T95I; G142-D140; G339-D336; S371-L368; S373-P370; S375-F372; G446-S443; R452-L449; E484-A481; G496-S493; N501-Y498; Y505-H502; H655-Y652; D796-Y793; E1238-G1235; L1240-S1237; K1247-L1241; D1248-F1242; D1250-G1244; D1251-P1245; K1252-G1246	G142-D140; N211-I206; G339-D336; S371-L368; S373-P370; S375-F372; G446-S443; E484-A481; G496-S493; N501-Y498; Y505-H502; H655-Y652; A682S; D793Y;

Parameters which used in different server:

For SuperPose version 1.0:

Alignment Options:

PDB Entry A: Restrict superposition to residues: **All**

PDB Entry B: Restrict superposition to residues: **All**

Secondary Structure Alignment:

Guide the superposition with a secondary structure alignment rather than a sequence alignment when pairwise sequence identities fall below**: 25%**

Subdomain Matching:

SuperPose can look for structurally similar and dissimilar regions between aligned protein chains. This is useful in identifying hinge motions, mobile segments, etc. If SuperPose finds structurally dissimilar regions, it will superpose the structures based on the single longest structurally similar region shared by the sequences.
Subdomain matching: **On**
Minimum Sequence Similarity:
Look for subdomain matches and mismatches (e.g. hinge regions) for sequences with pairwise sequence identities above: **80%**
Similarity Cutoff:
Identify as 'similar' aligned alpha-carbon atoms with RMSDs less than **2 Angstroms.**
Dissimilarity Cutoff:
Identify as 'dissimilar' aligned alpha-carbon atoms with RMSDs greater than **3.0 Angstroms**
Dissimilar Subdomain:
The minimum number of contiguous alpha-carbon atoms with RMSDs above the Dissimilarity Cutoff (above) required to be considered a 'dissimilar' subdomain is **7 atoms.**

Table S2. Parameter for HADDOCK 2.4.

**Parameters**	**Easy interface**
Active and passive residue lists	Supported
Docking from ensemble structures	Supported
Protein–nucleic acid docking	Supported
Cofactors and modified amino acids	Supported
Semi-flexible segment definition	Automatic
Fully flexible segment definition	No
Histidine protonation states	Automatic
Custom CNS distance restraints	No
Custom hydrogen bond restraints	No
Custom dihedral angle restraints	No
Preservation of nucleic acid base pairing and backbone conformation	Automatic
Ab initio docking	No
Nonpolar hydrogens	No
Random removal of restraints	Automatic
Number of structures to dock and to refine	Automatic
DMSO refinement	No
Solvated docking	No
Epsilon (electrostatic scaling constant)	Automatic
Clustering	Automatic
Symmetry	No
Restraints energy constants	Automatic
Relaxation anisotropy restraints	No
Residual dipolar couplings	No
Energy and interaction parameter	Automatic
Scoring parameters	Automatic
Randomization of starting orientations	Automatic
Refinement-only protocol	No
Temperature and time steps of the various refinement stages	Automatic

For PRODIGY:

Temperature (in °C): By default the value of the dissociation constant (K_d) is calculated at 25 °C. The user can change this value to any desired temperature.
